# Properties of Electrode Induction Melting Gas Atomization- and Vacuum Induction Melting Atomization-Produced Powders and Their As-HIPed Blanks

**DOI:** 10.3390/ma18030710

**Published:** 2025-02-06

**Authors:** Xiaona Ren, Yao Wang, Zhenfan Wang, Peng Wang, Zihao Wang, Lebiao Yang, Weifeng Qi, Xinggang Li, Changchun Ge

**Affiliations:** 1 School of Materials Science and Engineering, University of Science and Technology Beijing, Beijing 100083, China; 2 Shenzhen PICEA Robotics Co., Ltd., Shenzhen 518107, China; 3 China Machinery Institute of Advanced Materials (Zhengzhou) Co., Ltd., Zhengzhou 450001, China; 4 Department of Materials Science and Engineering, Southern University of Science and Technology, Shenzhen 518055, China

**Keywords:** powder metallurgy, superalloy, VIGA, EIGA, HIP

## Abstract

The main method for large-scaled preparing powder superalloys in the production process is inert gas atomization, particularly vacuum-induced gas atomization (VIGA). A novel technique called electrode-induced gas atomization (EIGA) with a crucible-free electrode was proposed to prepare non-inclusion superalloy powders. In this study, a Ni-based superalloy of FGH4096 powder was prepared using both the VIGA and EIGA methods, while blanks were prepared through direct hot isostatic pressing (as-HIPed) near-net-forming method. The particle size, morphology, microstructure, and mechanical properties of the powders and blanks were compared via a laser particle size analyzer, SEM, TEM, and room-temperature and 650 °C tensile tests. The results indicated that EIGA-prepared powders exhibited a finer particle size and better surface quality than the one prepared via VIGA, which showed reduced satellite powders. However, the as-HIPed blank of EIGA-prepared powders had a lower secondary γ’ ratio and slightly reduced strength compared to the as-HIPed blank of VIGA-prepared powders due to its slightly lower secondary γ’ phase ratio and less effective inhibition of dislocation movement. Furthermore, the overall performance of the two samples did not differ significantly due to the similar microstructural characteristics of the powders. However, the variation in particle size affects heat conduction during the HIP process, resulting in slight differences in blanks’ properties.

## 1. Introduction

The powder metallurgy (PM) technique is widely utilized in the aerospace engine manufacturing field for the preparation of superalloy components, since the PM technique can fabricate components with a short process and low cost [1]. Direct hot isostatic pressing (as-HIP) represents a significant PM technology aimed at fabricating parts with intricate geometries and unworkable characteristics. As-HIPed parts demonstrate a near net shape, homogeneous structure, high density or full density, eliminated porosity, and cost-effectiveness, since the spherical powders experience a gradual reduction in interparticle pores with the synergistic effects of elevated temperature and pressure, followed by particle rearrangement, plastic deformation, and diffusion creep, ultimately leading to successful compaction and sintering [2,3,4,5,6]. However, HIPed superalloys invariably exhibit prior particle boundaries (PPBs), which can significantly compromise their mechanical properties. Fortunately, our research has demonstrated that refining the size of these PPBs may mitigate these adverse effects [7]. Hence, the as-HIP process is highly suitable for fabricating aeroengine components with minimal macro-segregation and microstructure heterogeneity [8,9,10].

The inert gas atomization (IGA) technique is extensively utilized for the preparation of metal or alloy powders, particularly those used in 3D printing and aerospace superal-loys. This process enables the rapid solidification of powders and facilitates the incorpo-ration of appropriate strengthening elements [11]. Importantly, the IGA technique produc-es fine particle sizes and highly spherical powders [12], which are crucial for PM superalloy components. Vacuum induction melting atomization (VIGA) is widely recognized as a high-performance method for fabricating metal/alloy powders due to its advantages, including high sphericity, fast cooling rate, low oxygen content, and suitability for various metals and alloys [13]. This technology primarily disrupts the downward flow of molten metal from the smelting chamber through the application of supersonic, high-kinetic-energy inert atomizing gas, ultimately cooling and solidifying it into spherical metal powder. Currently, VIGA stands as one of the most widely utilized techniques for the preparation of superalloy powders such as K417G [14], Inconel 718 [13], GTD222 [15], Haynes230 [16], FGH96, and IN625 [17]. The as-prepared superalloy powders are mainly applied for additive manufacturing, owing to their high quality and properties, as aforementioned. As another significant IGA technique, EIGA (electrode induction melting gas atomization) represents a crucible-free technology for preparing metal powders suitable for producing ultra-clean, refractory, and reactive alloy powders such as Mo, Ti, Nb, and Zr [18,19,20]. This technology entails the creation of a continuous and precisely con-trolled molten liquid flow at the lower end of the alloy bar, facilitated by the melting effect of the electrode induction coil. The supersonic nozzle subsequently atomizes the molten alloy stream into fine metal droplets using a high-speed argon gas flow, which are then cooled to form metal powder. A key feature of this process is that the melt remains in a contact-free state with any ceramic crucible throughout, thereby preventing the introduction of ceramic inclusions during powder preparation.

Ni-based superalloys are widely utilized in aerospace engine disks [21], while they face challenges related to PPBs, thermally induced porosity (TIP), and inclusions. Although improvements have been made regarding PPBs and TIP, the presence of inclusions remains a significant issue. Inclusion will result in a reduction in its mechanical properties, particularly its fatigue resistance [22,23,24], which primarily originates from ceramic contaminants within the powder. To reduce the size of these ceramic inclusions, it is necessary to either decrease the particle size of the powder or employ powder preparation techniques that do not involve contact with refractory materials. To address this problem, our research group has developed a Ni-based superalloy powder preparation technique using EIGA that enables the production of high-purity, highly spherical FGH4096 superalloy (the second generation of Ni-based superalloys in China) powders with smooth surfaces and no hollow particles among sizes below 150 μm. Furthermore, this process eliminates non-metallic inclusions [25,26]. The resulting FGH4096 superalloy powders exhibit high purity, excellent sphericity, smooth surfaces, the absence of hollow particles within sizes below 150 μm, and no additional inclusions introduced during the powder preparation process [26]. Unlike VIGA, which is extensively utilized in the field of superalloy powder preparation, powder superalloys produced through the EIGA process have a relatively shorter history and a more limited variety. In addition to FGH4096 superalloy powder, Inconel 718 alloy and Rene 95 alloy powders have also been successfully prepared using EIGA and formed into exceptional components through HIP [3,13]. With the advancement of additive manufacturing technology, superalloy powders produced by either the EIGA or VIGA process are predominantly formed through additive manufacturing techniques for researches. The as-HIP technique, however, remains less commonly utilized, with the exception of Russia, where this forming method is predominant.

In this study, we investigated the correlation between powder quality and the properties of HIPed components fabricated from superalloy FGH4096. The powders utilized in this investigation were prepared using both the VIGA and EIGA methods. The differences were analyzed, which were particle size, powder morphology, and the precipitated strengthened phase and mechanical properties of the HIPed blanks.

## 2. Experiments

### 2.1. Powder Fabrication and Characterization

**Figure 1:**
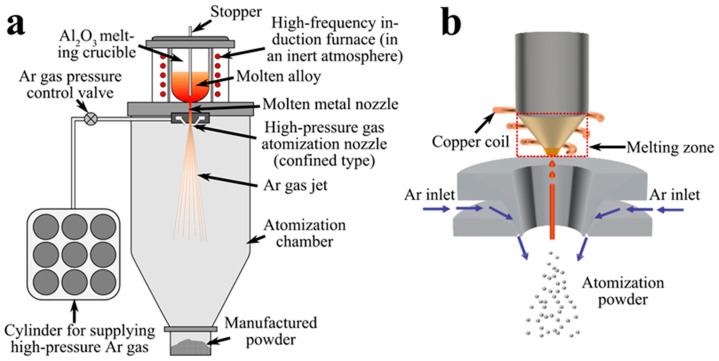
Mechanism illustration of the two techniques: VIGA [11] (**a**) and EIGA [27] (**b**).

FGH4096 powders were prepared via VIGA (Shenzhen Wedge Aviation Technology Co., Ltd., Shenzhen, China) and EIGA (Anhui Yingyuan New Material Technology Co., Ltd., Ma’anshan, China) at 4 Pa, respectively. As depicted in Figure 1, the VIGA process involves storing the molten alloy in a ceramic crucible, which may introduce non-metallic inclusions. Conversely, the EIGA process atomizes the alloy directly by passing it through a copper coil. The specific powder production process entails positioning the master alloy within a melting crucible during the VIGA process or an induction coil for the EIGA process. High-purity argon gas is introduced to maintain a slight positive pressure environment. The master alloy is subsequently heated until it reaches a molten state, after which it is transferred to an intermediate ladle and atomized into powder. The morphology of the as-prepared powders was examined using SEM and EDS with a JSM-6701F scanning electron microscope, while powder size analysis was conducted using a Mastersizer 3000 laser particle size analyzer.

### 2.2. Blank Preparation and Characterization

The obtained powders, with sizes ranging from 50 to 100 μm, were encapsuled in Q235 low-carbon steel with the same process as [28] and then underwent HIP at a temperature of 1180 °C (with 150 min from room temperature) and pressure of 150 MPa for a duration of 3 h, using a Sweden Quintus QIH-9 device and furnace cooling. The resulting blanks had dimensions measuring 85 mm × 141 mm and were designated as VB (VIGA-prepared powders HIPed blank) and EB (EIGA powders HIPed blank). The FGH4096 blanks acquired thus were analyzed using a Nordly Max2 Field Emission Scanning Electron Microscope and HKL Technology Channel 5 for EBSD analysis (Oxford instruments, Abingdon, UK), as well as SEM (Zeiss GeminiSEM 500 outfitted with an In-lens detector, Carl zeiss AG, Oberkochen, Germany) for the examination of fracture morphology and microstructure. Tensile properties at room temperature (RT) and at 650 °C were evaluated using the Jinan Test Gold Group Co., LTD WDW200D (Jinan, China) and Changchun Machinery Factory (Changchun, China) DDL-50 electronic universal testing machine, respectively [28].

## 3. Results and Discussion

### 3.1. Results and Discussion of the as Prepared Powders

**Figure 2:**
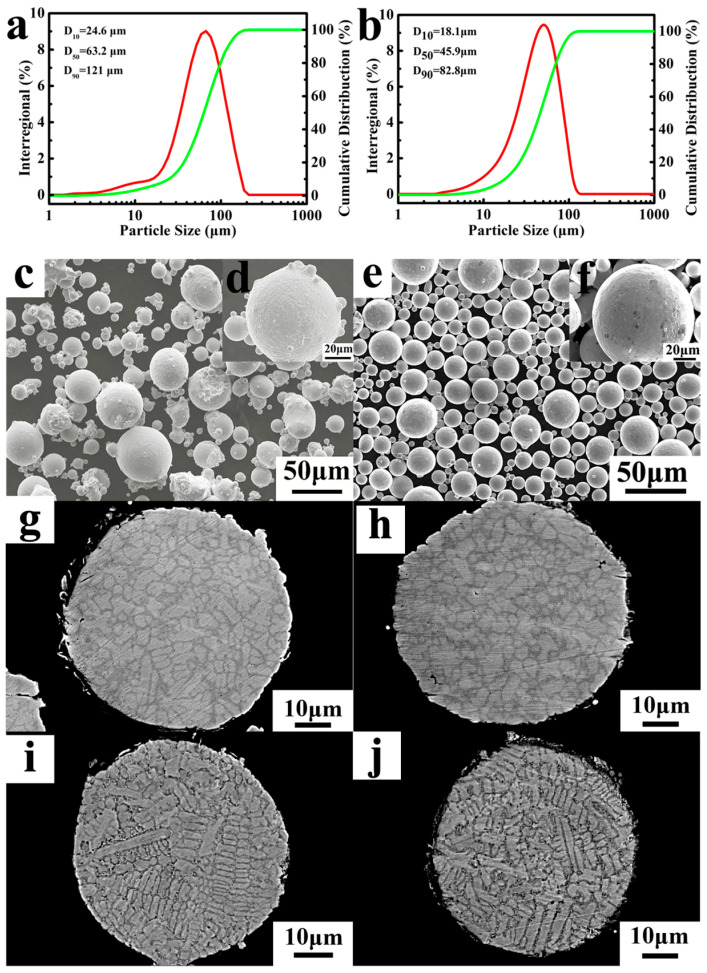
Features of the as-obtained powders: powder size distribution (**a**,**b**) and morphology (**c**–**j**) of VIGA- (left) and EIGA (right)-prepared FGH4096 superalloy powders.

The quality of FGH4096 superalloy powder prepared by EIGA was superior to that prepared by VIGA. Firstly, EP exhibited a finer particle size (with D50 and D90 of 45.9 μm and 82.8 μm, respectively) compared to VP (with D50 and D90 of 63.2 μm and 121 μm, respectively) (Figure 2a,b). During the EIGA process, the droplets were consistently discontinuous, whereas they remained continuous during the VIGA process [12]. Therefore, the higher vapor–liquid ratio in EIGA resulted in finer powders, with the discontinuous droplets contributing to this fine powder formation [29].

**Figure 3:**
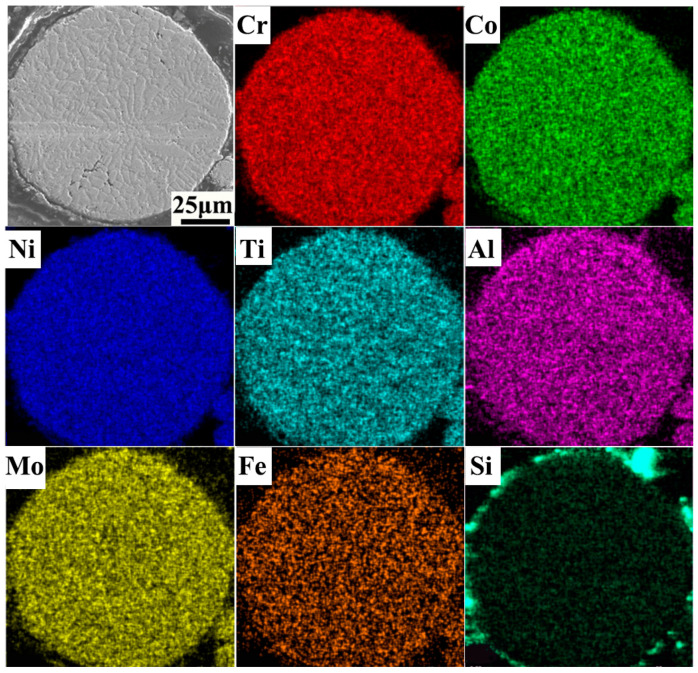
Elemental maps of the powders prepared by VIGA.

**Figure 4:**
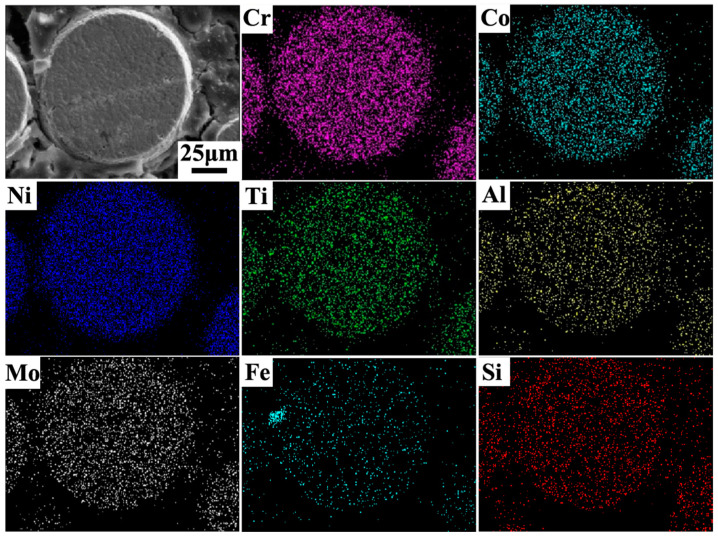
Elemental maps of the powders prepared by EIGA.

Secondly, in comparison to VP, the EP exhibited higher sphericity and a reduced presence of satellite powder (Figure 2c–f). The formation of satellite powder primarily arises from the collision between solidified fine particles and unsolidified coarse particles within the atomizing furnace’s dust circle [30]. During the EIGA process, the discontinuous droplets are effectively disrupted by high-speed gas jets, thereby minimizing dust rotation within the atomizing furnace due to lower levels of discontinuous melt and inhibiting collisions between solidified particles and droplets. Conversely, owing to its higher atomization efficiency, it is inevitable that VIGA results in extensive collisions between fine particles and unsolidified droplets, leading to a significant generation of metallurgical satellite powders within the dust rotation zone. Consequently, EP exhibited a significantly lower incidence of satellite powders compared to VP, while both exhibited comparable microstructures due to the rapid solidification process of the powders. Additionally, satellite powders can significantly affect the flowability and compactability of the powder materials by forming large agglomerates or impeding proper packing. Furthermore, the primary constituents of FGH4096 superalloy powders, including Ni, Cr, Co, Ti, Al, Mo, Fe, and Si, are illustrated in Figure 3 (VIGA-prepared powder) and Figure 4 (EIGA-prepared powder). These figures demonstrate that the elements in both powders were distributed uniformly, without significant enrichment or depletion. This uniform distribution is another characteristic outcome of the rapid cooling process during atomization.

### 3.2. Results and Discussion of the HIPed Blanks

**Figure 5:**
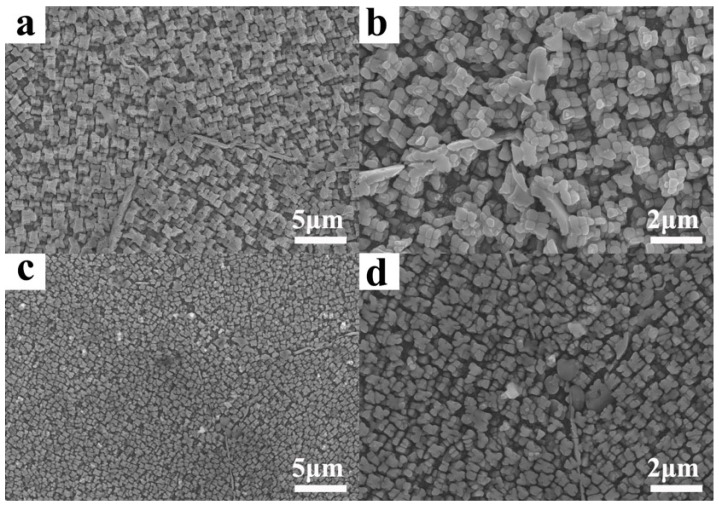
Scanning electron microscope images of HIPed blanks using VIGA-prepared powders (**a**,**b**) and EIGA-prepared powders (**c**,**d**).

**Figure 6:**
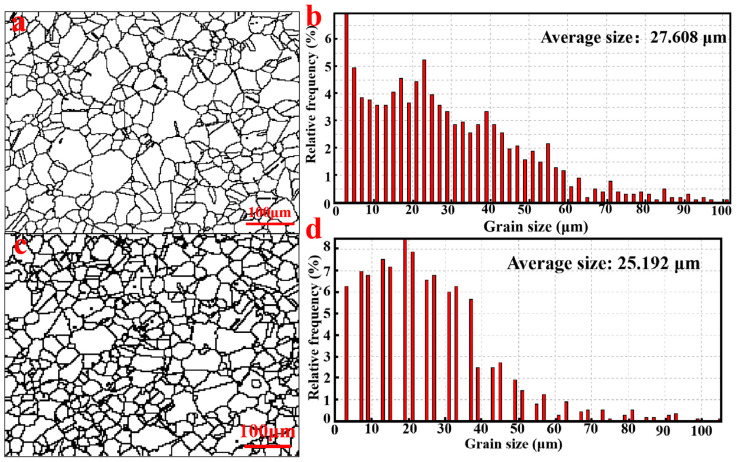
Grain size distribution of HIPed blanks using VIGA-prepared powders (**a**,**b**) and EIGA-prepared powders (**c**,**d**).

The phase composition of the blanks prepared using the two powders was identical, consisting of elongated or irregular primary γ′ phases, petal-like secondary γ′ phases, and small nearly spherical tertiary γ′ phases (Figure 5). However, slight differences were observed between these two samples regarding shape and size of their secondary γ′ phases. It is apparent that the γ′ phase sizes in VB were significantly larger than those in EB (Figure 5a,c and Figure 6).

**Figure 7:**
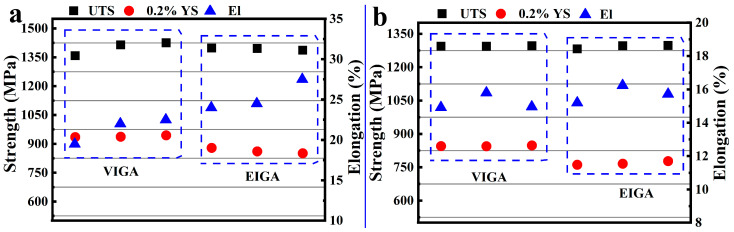
Tensile properties of HIPed blanks at room temperature (**a**) and 650 °C (**b**), which used VIGA-prepared powders and EIGA-prepared powders.

**Figure 8:**
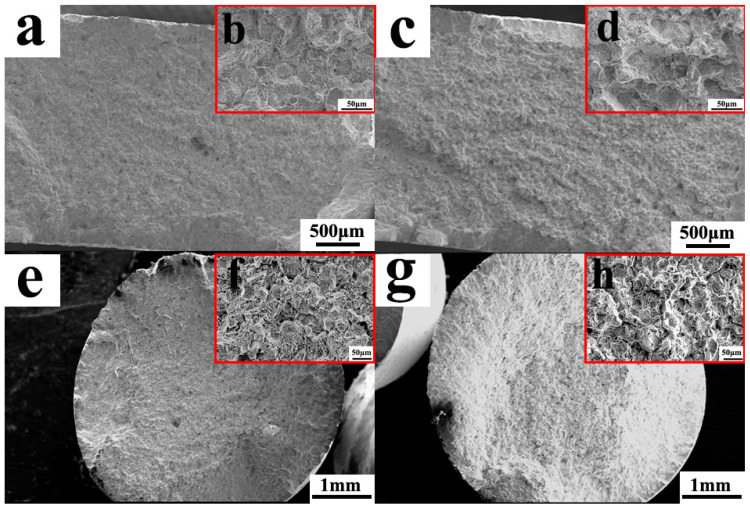
Scanning electron microscope images of HIPed blanks tensile fracture at room temperature (**a**–**d**) and 650 °C (**e**–**h**), which used VIGA-prepared powders (left) and EIGA-prepared powders (right).

The ultimate tensile strength of VB and EB at RT was very similar, as shown in Figure 7a (1399 ± 35.93 MPa and 1393.67 ± 35.93 5.86 MPa, respectively). However, the yield strength of VB (938.67 ± 4.73 MPa) slightly surpassed that of EB (863.67 ± 14.19 MPa), while the ductility of VB (21.33 ± 1.61%) was marginally lower than that of EB (25.33 ± 1.89%). It is well established that superalloys prepared from fine powder exhibit enhanced properties due to refined crystalline strengthening compared to those prepared from coarse powder [7]. The fracture morphology at RT of the VB and EB samples is shown in Figure 8a–d. Both processes resulted in mixed fractures involving toughness and brittleness. However, transgranular fractures occurred less frequently in EB, indicating higher plasticity.

Comparing tensile properties, the results of 650 °C tensile testing were consistent with those obtained from RT tensile testing (Figure 8e–h). Specifically, both VB and EB exhibited similar ultimate tensile strengths (1294.67 ± 1.4 MPa and 1291.94 ± 8.61 MPa, respectively), while the yield strength of VB (845.92 ± 2.09 MPa) surpassed that of EB (768.38 ± 8.35 MPa), and the ductility of VB (15.23 ± 0.5%) slightly lagged behind that of EB (15.72 ± 0.52%). The 650 °C tensile fractures of VB (Figure 8e,f) and EB (Figure 8g,h) exhibited an increased presence of transgranular fracture surfaces compared to the RT samples, with relatively fewer intergranular fractures observed. With increasing temperature, both the grain interior and boundary experienced strength reduction. The reduced occurrence of intergranular fractures suggested that 650 °C is not an equal strength temperature for both the grain interior and the boundary. Under this temperature, the grain interior became weaker than the grain boundary. Consequently, both VB and EB displayed mixed ductile–brittle fracture characteristics, with a combination of intergranular fractures and dimple fractures in both samples. Furthermore, since VB and EB exhibited similar numbers and distributions of transgranular fracture surfaces under 650 °C conditions, they demonstrated closed plasticity.

**Figure 9:**
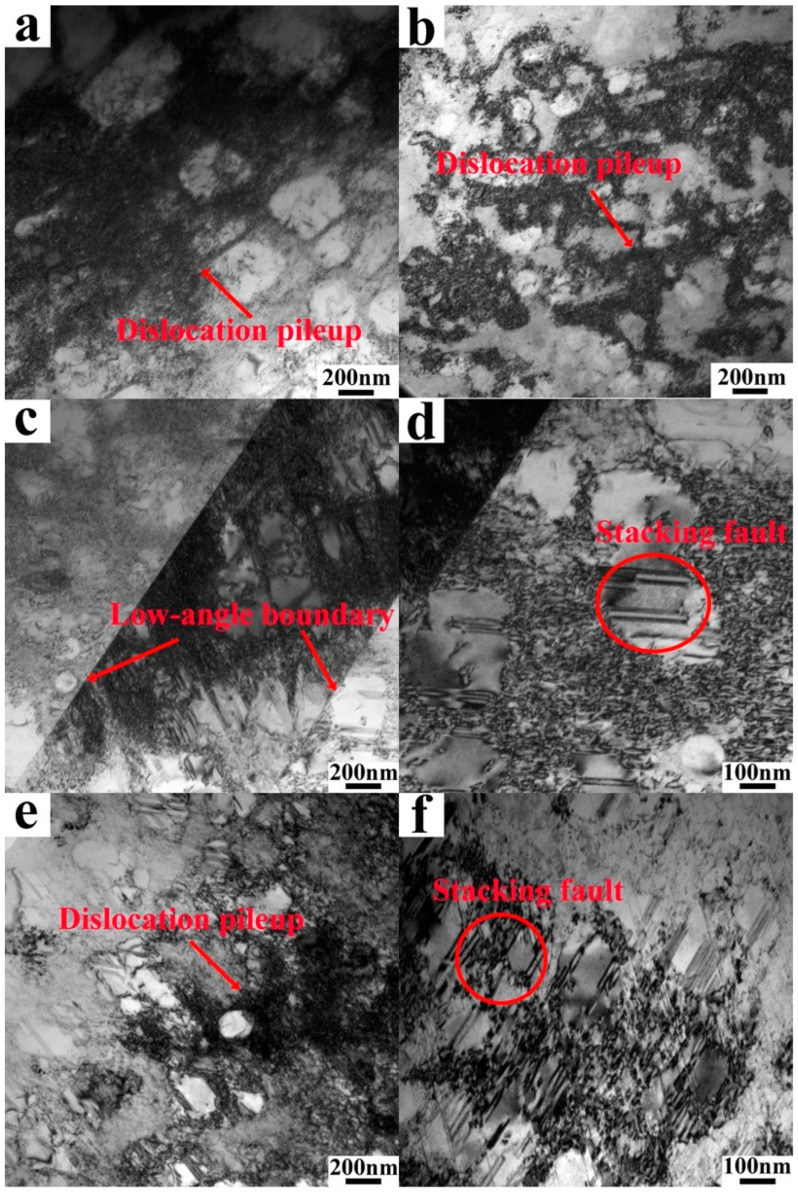
TEM of HIPed blank tensile samples at room temperature (**a**,**b**) and 650 °C (**c**–**f**), using VIGA- (left) and EIGA-prepared powders (right).

In the region located 1 mm below the tensile fracture at room temperature of both VB and EB, dislocation dominated in the region (Figure 9a,b). Serious dislocation entanglements occurred at the grain boundaries, while small grain sizes impeded dislocation motion. In the same region of the tensile fracture at 650 °C (Figure 9c–f), layered faults, stacking faults, and dislocations dominated both VB and EB samples. Similar to the room-temperature samples, severe dislocation blockage occurred at the grain boundaries. Additionally, the grain boundaries exhibited an intertwining of dislocations and layering faults, while stacking faults were predominantly found within the grains, as observed in Figure 9. Notably, in Figure 9c, distinct grain boundaries with small angles are evident. However, no discernible grain boundaries were observed in EB, as depicted by Figure 9e. Furthermore, stacking faults with a concentrated distribution density primarily constituted the intermediate-level faults in EB (Figure 9f).

### 3.3. Strengthening Mechanism of the HIPed Blanks

**Figure 10:**
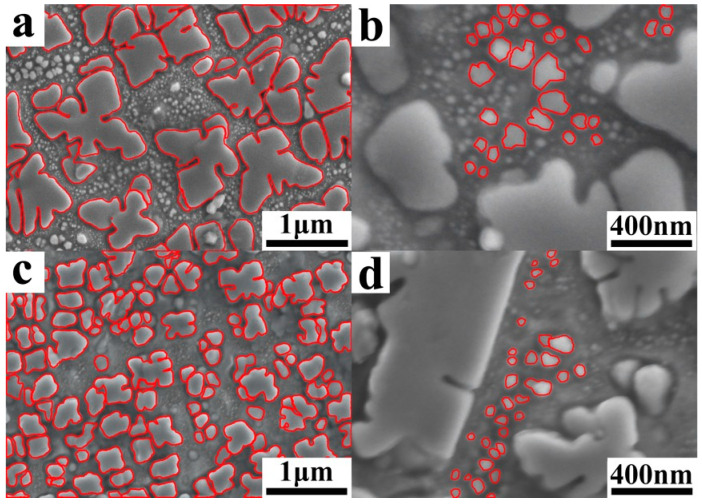
Scanning electron microscope images of the size and morphology of the secondary γ’ phase in HIPed blanks, using VIGA-prepared powders (**a**,**b**) and EIGA-prepared powders (**c**,**d**).

**Figure 11:**
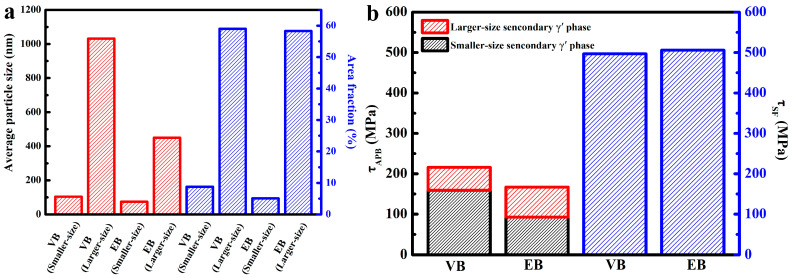
The average particle size and area fraction of the secondary γ’ phase in HIPed blanks (**a**) and the values of τ_APB_ and τ_SF_ of the two HIPed blanks (**b**), using VIGA-prepared powders and EIGA-prepared powders (marked as VB and EB, respectively).

In nickel-based superalloys, the γ′ phase is the main strengthening phase, especially the secondary γ′ phase. The microstructure of the secondary γ′ phase in the two samples is illustrated in Figure 10. The average particle size and area of these secondary γ′ phases were determined assuming a spherical shape for each particle. The resulting mean particle sizes are presented in Figure 11a. It can be observed that both the larger-size and smaller-size secondary γ′ phases of VB demonstrated significantly larger average particle sizes compared to those of EB, which aligned with our previous findings. Furthermore, there was no significant difference in the area fraction occupied by the larger-sized secondary γ′ phase between the two samples. However, EB showed a lower area fraction for the smaller-sized secondary γ′ phase due to the smaller dimensions of its larger-sized counterpart facilitating the absorption of smaller particles under high-temperature and -pressure conditions.

The essence of antiphase boundary (APB) shear at room temperature and stacking fault (SF) shear at high temperatures is the strengthening of the secondary γ′ phase [31,32]. Therefore, we calculated the τ_APB_ and τ_SF_ of the two HIPed blanks, with the same detailed calculation process as [28], and the result is shown in Figure 11b.

The as-obtained results demonstrated a consistent correlation between the calculated values (Figure 11b) and experimental data (Figure 7). Specifically, at RT, VB exhibited a slightly higher strength value compared to EB, whereas at high temperatures, they exhibited similar characteristics of tensile strength. An analysis of Figure 11b revealed that the disparity in the area fraction of smaller-sized γ′ phase secondary precipitation is the primary factor contributing to the divergence in RT performance between the two blanks. Notably, the influence of the area fraction pertaining to the secondary precipitation γ′ phase on performance becomes more pronounced at RT, while at elevated temperatures, it is contingent upon the overall proportion of secondary precipitation γ′ phase. The analysis of the morphological characteristics of powders and blanks indicated that, during the HIP process, the microstructural characteristics of the powders have minimal influence on the blanks. In contrast, particle size has a more significant impact due to its effect on thermal conductivity.

## 4. Conclusions

Powders prepared by VIGA and EIGA were consolidated into blanks through HIP. Subsequently, the microstructure, tensile properties, and fracture properties of the HIPed blanks were analyzed. Among the utilized powders, EIGA-prepared powders exhibited a smaller particle size (D50 of 45.9 μm) and superior surface quality compared to VIGA-prepared powders, which had larger particles (D50 of 63.2 μm) and more surface satellite powder. Following HIP forming, it was observed that the secondary precipitated γ′ in consolidated blank samples with EIGA-prepared powders possessed a finer grain size than that in consolidated blank samples with VIGA-prepared. Additionally, the total size of secondary precipitated γ′ in consolidated blank samples with EIGA-prepared powders was slightly smaller than that in consolidated blanks with VIGA-prepared powders. These differences primarily account for their disparate performance at room temperature. Furthermore, it should be noted that the genetic characteristics of the microstructure within the powder barely influence the performance of hot isostatic compacts, while the particle size has more influence on it. Regarding the mechanical properties, the room-temperature yield strength of consolidated blanks with VIGA-prepared powders (938.67 ± 4.73 MPa) exceeded that of consolidated blanks with EIGA-prepared powders (863.67 ± 14.19 MPa), whereas the room-temperature ductility of consolidated blanks with VIGA-prepared powders (21.33 ± 1.61%) was marginally lower than that of consolidated blanks with EIGA-prepared powders (25.33 ± 1.89%). The trend observed in high-temperature tensile results aligned with that of room-temperature tensile results. Additionally, the fracture surfaces of both processes at both room and high temperatures exhibited a combination of ductile and brittle fractures. TEM revealed a significant number of dislocation pile-ups at grain boundaries, along with a considerable presence of stacking faults. In the as-HIPed alloys, the secondary γ′ phase serves as the primary strengthening phase, employing the APB shear strengthening mechanism at room temperature and the SF shear strengthening mechanism at elevated temperatures.

## Data Availability

The original contributions presented in this study are included in the article. Further inquiries can be directed to the corresponding author.
